# Comparison of Laryngoscopic Views between C-MAC™ and Conventional Laryngoscopy in Patients with Multiple Preoperative Prognostic Criteria of Difficult Intubation. An Observational Cross-Sectional Study

**DOI:** 10.3390/medicina55120760

**Published:** 2019-11-27

**Authors:** Aikaterini Amaniti, Panagiota Papakonstantinou, Dimitrios Gkinas, Ioannis Dalakakis, Evangelia Papapostolou, Anastasia Nikopoulou, Marianna Tsatali, Paul Zarogoulidis, Konstantinos Sapalidis, Christoforos Kosmidis, Charilaos Koulouris, Dimitrios Giannakidis, Konstantinos Romanidis, Panagoula Oikonomou, Nikolaos Michalopoulos, Aris Ioannidis, Kosmas Tsakiridis, Anastasios Vagionas, Isaak Kesisoglou, Vasilios Grosomanidis

**Affiliations:** 1 Department of Anesthesia and Critical Care, AHEPA University Hospital, Aristotle University of Thessaloniki, 541 24 Thessaloniki, Greece; amanitik@gmail.com (A.A.); tsapap@gmail.com (P.P.); pkamparoudi@outlook.com (D.G.); idalakakis@gmail.com (I.D.); vtzarou@yahoo.com (E.P.); ggalaktidou@yahoo.com (A.N.); vgrosoma@auth.gr (V.G.); 2 Psychology Department, The University of Sheffield International Faculty, City College, 546 26 Thessaloniki, Greece; stellalaskou1981@gmail.com; 3 3rd Department of Surgery, “AHEPA” University Hospital, Aristotle University of Thessaloniki, Medical School, 541 24 Thessaloniki, Greece; sapalidiskonstantinos@gmail.com (K.S.); dr.ckosmidis@gmail.com (C.K.); charilaoskoulouris@gmail.com (C.K.); giannakidis.d@gmail.com (D.G.); nmichalopoulos1@outlook.com (N.M.); stellalaskou@outlook.com (A.I.); isaackesisoglou@outlook.com (I.K.); 4 Second Department of Surgery, University Hospital of Alexandroupolis, Medical School, Democritus University of Thrace, 68100 Alexandroupolis, Greece; romanidis@yahoo.com (K.R.); pen.ek@hotmail.com (P.O.); 5 Thoracic Surgery Department, “Interbalkan” European Medical Center, 555 35 Thessaloniki, Greece; kosjohn@otenet.gr; 6 Oncology Department, General Hospital of Kavala, 655 00 Kavala, Greece; tvagionas@yahoo.com

**Keywords:** laryngoscopy, c-mac, intubation, conventional laryngoscopy

## Abstract

*Background and Objectives:* Video laryngoscopy has been proven useful under difficult airway scenarios, but it is unclear whether anticipated improvement of visualization is related to specific difficult intubation prognostic factors. The present study evaluated the change in laryngoscopic view between conventional and C-MAC^®^ laryngoscopy and the presence of multiple difficult intubation risk factors. *Materials and Methods:* Patients scheduled for elective surgery with >2 difficult intubation factors, (Mallampati, thyromental distance (TMD), interinscisor gap, buck teeth, upper lip bite test, cervical motility, body mass index (BMI)) were eligible. Patients underwent direct laryngoscopy (DL) followed by C-MAC™ laryngoscopy (VL) and intubation. Change of view between DL and VL, time for best view, intubation difficulty scale (IDS) and correlation between prognostic factors, laryngoscopic view improvement, and IDS were measured. *Results*: One-hundred and seventy-six patients completed the study. VL lead to fewer Cormarck–Lehane (C/L) III-IV, compared to DL (13.6% versus 54.6%, *p* < 0.001). The time to best view was also shorter (VL: 10.82 s, DL: 12.08 s, *p* = 0.19). Mallampati III-IV and TMD ≤ 6 cm were related to improvement of C/L between DL and VL. Logistic regression showed these two factors to be a significant risk factor of the glottis view change (*p* = 0.006, AUC-ROC = 0.57, 95% CI: 0.47–0.66). 175/176 patients were intubated with VL. 108/176 were graded as 0 < IDS ≤ 5 and 12/176 as IDS > 5. IDS was only correlated to the VL view (*p* < 0.0001). *Conclusion*: VL improved laryngoscopic view in patients with multiple factors of difficult intubation. Mallampati and TMD were related to the improved view. However, intubation difficulty was only related to the VL view and not to prognostic factors.

## 1. Introduction

While direct laryngoscopy (DL) with the conventional Macintosh laryngoscope still remains the most common technique for endotracheal intubation, the introduction of video-assisted laryngoscopy (VL) during recent years has changed that practice. Video laryngoscopes have designed to overcome some of the challenges associated with the difficult airway. The technique is considered one of the major advances in anesthesia practice during recent years [1]. The role of VL in difficult intubation scenarios, has recently been recognized in both ASA and DAS guidelines [2,3]. The DAS 2015 guidelines recommend that all anesthetists should be trained in video laryngoscopy and have immediate access to a video laryngoscope at all times.

Today, there is a number of commercially available video laryngoscopes with advantages or disadvantages, depending on the situations the clinicians have to manage [4]. Among them, the Storz^®^ C-MAC™ (Karl Storz GmbH & Co. KG, Tuttlingen, Germany) was the first Macintosh-type laryngoscope, incorporating the features of a conventional and video laryngoscope in one device. During the former case, the manipulation of the blade to the oral and pharyngeal structures and the technique of laryngoscopy is identical to conventional Macintosh blade laryngoscopy. However, when conventional laryngoscopy results in poor glottis view, due to inability to align the oral, pharyngeal, and laryngeal axes, C-MAC™ can be used as a video laryngoscope. In this case, a midline insertion technique, without sweep of the tongue, can achieve an unobstructed view of the larynx. The latter can be achieved thanks to a 320 × 240 pixel small CMOS camera chip with an embedded optical lens, located in the distal part of the steel blade, offering a 60° angle of view in the vertical axis and 80° field of view in the horizontal axis [5].

The performance of Storz^®^ C-MAC™ has been studied in a series of RCT, examining both normal and difficult airway scenarios. However, while most clinically available laryngoscopes, including Storz^®^ C-MAC™, have been proven to significantly improve the laryngoscopic view, concerns have been raised regarding the ability of VL to reliably translate this improved view to successful tracheal intubation. However, current evidence has shown that video-laryngoscopes can improve both laryngoscopic view and intubation success in different airway scenarios. A recent meta-analysis, including 16 articles confirmed that in difficult airways, the Storz^®^ C-MAC™ showed superior success rates, glottic visualization, and less external laryngeal manipulations, compared to the Macintosh [6]. Nevertheless, despite different video laryngoscopes’ “success story”, including Storz^®^ C-MAC™, the problem of failed intubation could not be fully eliminated. Up to know, the technique of awake fiberoptic intubation under local anesthesia and mild sedation for the management of an anticipated difficult intubation is considered the safest [2,7].

At the same time, the choice between awake fiberoptic intubation and intubation under general anesthesia is still quite subjective. Apart from cases with known airway pathology or history of known difficult intubation, no preoperative diagnostic tool has been proven to be highly accurate, in order to help clinicians to decide on the best airway management plan. A meta-analysis of bedside screening tests, conducted by Shiga et al., found that the most common used tests, i.e., the Mallampati classification, thyromental distance (TMD), sternomental distance, mouth opening, and Wilson risk score yielded poor to moderate sensitivity (20–62%) and moderate to fair specificity (82–97%). The most useful bedside test for prediction was found to be a combination of the Mallampati classification and thyromental distance [8].

Nevertheless, available data regarding the sensitivity, specificity, and prognostic value of bedside screening tests, refers to conventional laryngoscopy. It is obvious that the technique of VL, enabling different conditions of glottis visualization possibly changes the accuracy of these tests, especially in cases of VL with curved blades [9,10]. However, it is possible that this is also true for Macintosh VL such as the Storz^®^ C-MAC™.

The aim of the present study was to compare the use of the Storz^®^ C-MAC™ video laryngoscope with conventional direct laryngoscopy in patients with known risk factors for difficult intubation during routine induction of anesthesia. Primary endpoints were the change of glottic visualization between direct laryngoscopy (DL) and the Storz^®^ C-MAC™ video laryngoscopy (VL). Co-primary endpoint was the evaluation of the correlation between preoperative difficult intubation criteria and improvement of visualization between DL and VL. Secondary endpoints were the time needed to achieve the best laryngoscopic view, the success rate of intubation with VL, the difficulty of intubation measured with intubation difficulty scale (IDS), and the correlation between prognostic difficult intubation criteria, IDS, and VL view.

## 2. Materials and Methods

The present prospective, observational, cross-over study was approved by the Institutional Review Board of AHEPA University Hospital, Aristotle University of Thessaloniki, Thessaloniki, Greece; identification number 13355/24-3-2015. All ASA I-III patients scheduled to undergo elective surgery under general anesthesia and requiring tracheal intubation that were preoperatively evaluated for the presence of possible risk factors for a difficult tracheal intubation. Patients were examined regarding (1) Mallampati score, assessed with the head in neutral position [11], (2) thyromental distance (TMD) [12], (3) interincisor gap (IIG) [13], (4) cervical spine motility [14], (5) presence of buck teeth, (6) upper lip bite test (ULBT) [15], and (7) body mass index (BMI). The presence of Mallampati score III or IV, TMD ≤ 6 cm, IIG ≤ 4 cm, the presence of buck teeth more than 0.5 cm, ULBT grade III and IV and > 15° reduction of cervical motility were considered as risk factors for difficult intubation. Furthermore, BMI was also measured in all patients. A cut-off of BMI > 30 kg·m^−2^ was chosen as a risk factor for difficult intubation [16].

Patients were possibly eligible for inclusion if they had two, three, or more of the predefined risk factors of difficult intubation. Exclusion criteria were age < 18 years, pregnancy, the presence of severe airway pathology (infectious or neoplastic), which would cause significant changes in the normal anatomic structures, the need for rapid sequence induction and known failed intubation in previous operations. All possibly eligible patients were informed about the nature, the scope, and the risks of interventions. Afterwards they participated in the study after written informed consent was obtained by each one.

All anesthetists participated in the study were experienced with more than two years’ experience in conventional laryngoscopy and at least six months experience in the use of Storz^®^ C-MAC™ video-laryngoscope. At the day of operation, patients were transferred to the operating table in a supine position. Their head was resting on a doughnut jelly and a pillow was put under their shoulders in order to set them in “sniffing position”. Standard monitoring was instituted including ECG, non-invasive blood pressure, pulse oximetry, BIS™ (Aspect Medical, Minneapolis, MN, USA), and muscle relaxation monitoring (Datex-Ohmeda E-NMT™, GE Healthcare, Helsinki, Finland), before induction of anesthesia. Afterwards, a venous cannula (18 Ga) was inserted in all patients and administration of lactated Ringer was started. Preoxygenation with high flow oxygen and facemask was performed for at least 5 min in order to achieve better oxygenation reserve. Induction of anesthesia was followed a standard protocol including remifentanil 0.3 μg·kg^−1^·min^−1^, propofol 2–3 mg·kg^−1^, and rocuronium 0.6 mg·kg^−1^. Induction of anesthesia was performed by an anesthetist, not involved in laryngoscopy and intubation. This anesthetist was also responsible for the safety of the patient regarding hemodynamic and respiratory parameters.

After loss of consciousness all patients were ventilated through a facemask. Mask ventilation was performed by the anesthetist responsible for laryngoscopy and intubation. If facemask ventilation was not considered sufficient (no thoracic elevation, no capnography tracing, suggesting adequate expiration or SpO_2_ < 95%) during this phase, patients were automatically excluded from the study and efforts were made towards establishment of adequate ventilation, according to difficult airway management algorithms.

After confirmation of good intubation conditions (adequate depth of anesthesia, no response to the train-of-four stimulation of the ulnar nerve) patients underwent laryngoscopy maneuvers. By design, all patients underwent two laryngoscopies, one with a conventional laryngoscope (DL) and one with a Storz^®^ C-MAC™ video laryngoscope (VL). In detail, the anesthetist responsible for laryngoscopy performed DL with conventional Macintosh blade #3. The performer was allowed to make any maneuvers in order to achieve the best laryngoscopic view, including BURP external laryngeal pressure, head movement, advancement or withdrawal of the blade, or increasing lifting force, but not changing the #3 Macintosh blade. After obtaining the best laryngoscopic view (DL view), the anesthetist performing the DL graded the best view using the modified version of the Cormack and Lehane (DL C/L) scoring system [17]. A class I, IIa, or IIb DL C/L view were considered as successful, while a C/L III or IV were considered as unsuccessful views. A third member of the team was responsible for recording the elapsed time for obtaining the best view, as well as all necessary external manipulations (DL time). This was defined as the time between the insertion of the blade through the teeth until the best visualization of the laryngeal opening. Afterwards, the patient was manually ventilated through a facemask for an extra period of 2 min. Again, if ventilation was not judged as adequate, the protocol was stopped, the patient was excluded from the study and an airway management plan, prompting adequate ventilation with no extra laryngoscopic efforts, was followed. When this 2 min ventilation period was completed uneventfully, the same operator performed laryngoscopy using a Storz^®^ C-MAC™ video laryngoscope with a classic #3 Macintosh blade (VL). Again, all necessary manipulations for obtaining the best possible view were permissible, including the selection between conventional insertion of the Macintosh blade with sweeping of the tongue or midline insertion without sweeping. The best Cormarck–Lehane view (VL C/L) and time to best view (VL time) were recoded, including external manipulations, using the same definitions mentioned above. Again, a class I, IIa, or IIb VL C/L view was considered as successful, while a VL C/L III or IV was considered as an unsuccessful view.

After recording VL C/L and VL time, the anesthetist attempted intubation of the patient. Any necessary aids (such as rigid stylet or soft bougie) were at his disposal for use and their usage was recorded in patient protocol. Finally, the number of intubation attempts through Storz^®^ C-MAC™ video laryngoscope and success rate were also recorded. Intubation attempt was defined as any advancement of the tube up to the glottic entrance, under continuous vision of the glottis. Successful intubation was confirmed with capnography tracings. Cuffed, 7.5 and 8.5 mm internal diameter endotracheal tubes were used for all female and male patients, respectively (except for 6.0 mm internal diameter, MLT microlaryngeal tube for microlaryngeal surgery). For ensuring patient safety, intubation attempts were limited to three. Upon failure, infusion of remifentanil was stopped and suggamadex 1.2 mg·kg^−1^ was administered, while the patient was ventilated through a facemask or laryngeal airway mask upon recovery from anesthesia.

### Statistical Analysis

Power analysis was used to identify the appropriate number of participants that should be recruited in the study. According to the GPower 3.0.10 (Heinrich-Heine-Universität Düsseldorf, Düsseldorf, Germany), with 88% power at 0.05 significance the sample size needed was 115. Statistical analysis was conducted by Statistical Package for the Social Sciences SPSS (Version 23.0) for Windows (SPSS Inc., Chicago, IL, USA).

Initially, the chi-square or the Fisher test was used to compare the proportion rates of DL C/L and VL C/L successful views. C/L grades I, IIa, and IIb were considered successful, whereas C/L views III or IV were considered as unsuccessful. Due to the fact that the laryngoscopic view was not normally distributed, we performed the non-parametric test of Wilcoxon. In more detail, the Wilcoxon-signed rank test was used for comparison between time elapsed for obtaining the best laryngoscopic view (DL time and VL time). Level of significance was set at a = 0.05.

Univariate analyses were performed in order to find possible relationships between difficult intubation prognostic risk factors and improvement of the laryngoscopic view from unsuccessful (C/L III or IV) to successful (C/L I, IIa, and IIb) between DL and VL. For that purpose, a dichotomous variable describing improvement (change from III or IV with DL to I, IIa, IIb with VL) was set. Standard cut-offs for risk factors were also used as follows: Mallampati classification 3 or 4, thyromental disease <6 cm, >15° cervical spine movement limitation, interincisor gap <4 cm, grade III and IV ULBT score, and >30 kg/m^2^ BMI score. Specifically, the chi-square test for categorical variables was used to test for relationships between the six aforementioned risk factors with the outcome variable, which is the improvement of the C/L scale which depicts the difference between the two different kinds of laryngoscopy (difference between DL C/L and VL C/L). Afterwards, nominal logistic regression was employed to identify the factors which were related with the outcome variable, by creating a two-category nominal variable, and could be therefore the best predictors of C/L scale’s decrease. Subsequently, the predictive ability of each risk factor was evaluated with the receiver operator characteristic (ROC) curve, in order to calculate its discriminant ability to predict the outcome variable. Pearson as well as Spearman correlations, for variables which were normal or not normal distributed, were also employed in order to identify the relationship between IDS with VL and prognostic risk factors, as well as the association between IDS with VL and VL views. Statistical analysis was conducted by the Statistical Package for the Social Sciences SPSS (Version 23.0) for Windows (SPSS Inc., Chicago, IL, USA), whereas the level of significance was set at a = 0.05.

## 3. Results

According to the GPower 3.0.10, with 88% power at 0.05 significance the sample size needed was 115 subjects, however a total of 178 patients met the criteria and gave informed consent to participate in the study from November 2017 to April 2019. Two patients (one man and one woman) were initially enrolled but finally excluded from the study. The first patient was a woman with Buck teeth test > 0.5 cm and class IV ULBT, scheduled for total thyroidectomy, who had very difficult mask ventilation. The protocol was stopped, the patient was ventilated with a fast-track LMA mask, and intubated through the mask. The second patient was a man with a Mallampati III and BMI > 30 kg/m^2^, scheduled to undergo cholecystectomy, who suffered severe bradycardia during conventional laryngoscopy. The patient was intubated through conventional laryngoscope and no more laryngoscopy attempts were made. Characteristics of the included patients are seen in Table 1.

### 3.1. Laryngoscopic View through DL and VL

**Figure 1:**
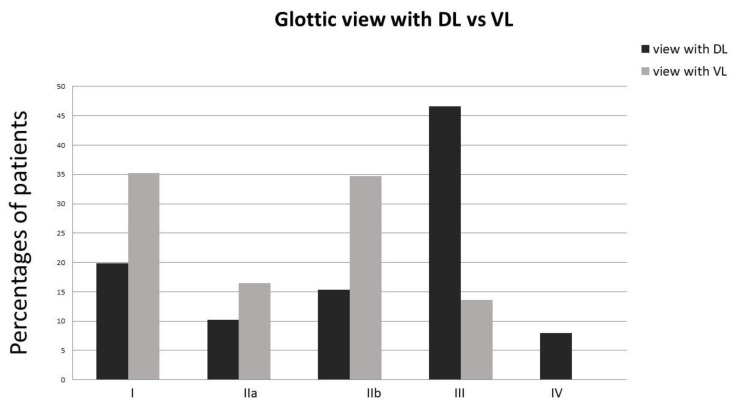
Percentages of patients with different C/L views under direct laryngoscopy (DL) and the Storz^®^ C-MAC™ video laryngoscopy (VL).

The percentages of patients with different C/L views for DL and VL are shown in Figure 1. When using the DL, unsuccessful view of the glottis (C/L grade III or IV) had a higher incidence (54.6%), whereas unsuccessful views under VL (C/L grade III, no C/L grade IV was observed) was significantly lower (13.6%) according to the chi-square test (*p* < 0.001). The mean best laryngoscopic view time was significantly higher for DL (12.08 s) in comparison to the time for VL (10.82 s, *p* = 0.019).

### 3.2. Relationship between Difficult Intubation Prognostic Risk Factors and Change of Laryngoscopic View

#### 3.2.1. Univariate Analysis

According to the univariate analysis (Table 2), no significant relationships were found between cervical spine movement, IIG gap, back teeth, ULBT score and BMI score, and the improvement of the Cormack–Lehane scale. However, Mallampati score III-IV was related to improvement of Cormack–Lehane (*p* = 0.006) with the VL. Moreover, there was evidence that the odds ratio of the TMD < 6 cm was found to be associated with improvement of the Cormack–Lehane view between DL and VL from III–IV to I–II (*p* = 0.05).

#### 3.2.2. Multivariate Model

**Figure 2:**
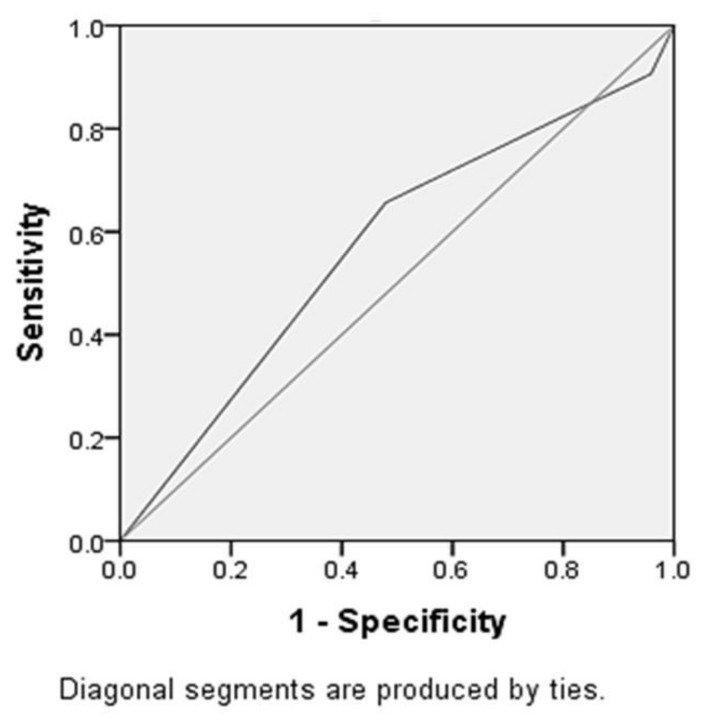
Receiver operator characteristic (ROC) of Mallampati-thyromental distance (TMD) binary index.

According to the univariate analysis, Mallampati airway and the thyromental distance, were independently associated with the better VL C/L view, compared to DL. Therefore, these two variables were included in the nominal logistic regression analysis to identify whether this model could predict the outcome measure. According to the results, this binary indicator model was a significant risk factor of the glottis view change (*p* = 0.006), with an area under the ROC curve of .57 (95% CI, 0.47, 0.66) (Figure 2).

#### 3.2.3. Intubation Difficulty with VL

Among the 176 patients who completed the predefined procedure with both DL and VL, all but one were successfully intubated with VL (success rate 99.4%). The only patient who was not intubated with VL had four risk factors of difficult intubation (Mallampati III, Thyromental < 6 cm, Buck teeth > 0.5 cm, and ULBT class IV). This patient has C/L III in both DL and VL. This case was recorded as failed intubation with the VL. However, the patient was finally blindly intubated with a conventional Macintosh #4 and an elastic boogie, without vision of the glottic inlet. Hence, he was recorded as IDS > 5.

**Figure 3:**
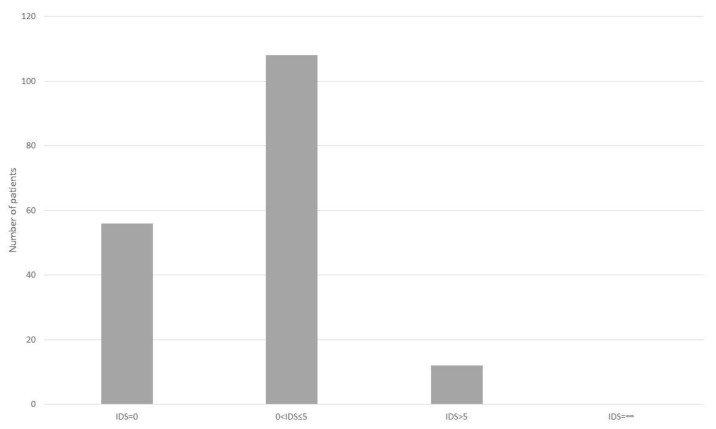
Number of patients with different IDS grades.

**Figure 4:**
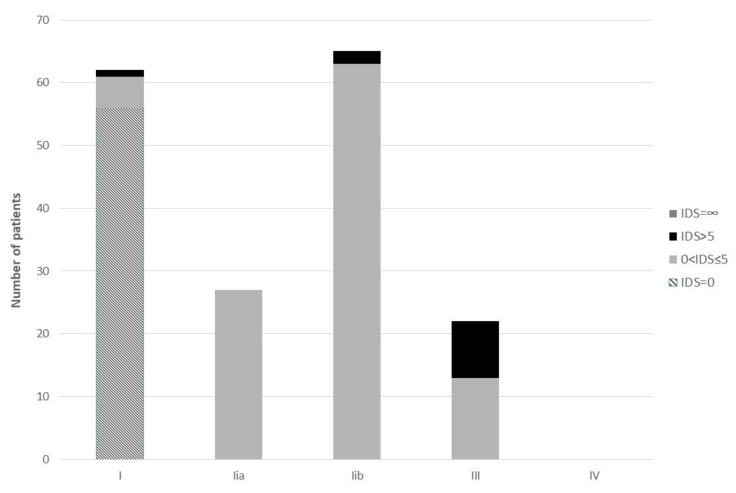
Correlation between VL laryngoscopic view and intubation difficulty scale (IDS).

Regarding the 175 successful intubations, 56 patients were graded as IDS = 0.108 with 0 < IDS ≤ 5 and 12 were graded as IDS > 5 (including the patient who finally intubated with the conventional #4 Macintosh). The correlation between VL laryngoscopic view and IDS is shown in Figure 3, Figure 4.

In total, 40 out of 176 patients were required more than one attempt to be intubated. In 12/176 cases, an extra attempt consisted of changing from classing insertion of VL blade with tongue sweeping to midline insertion. The remainder 28 out of 176 patients were intubated with the use of a gum elastic boogie. Nineteen of them had a C/L grade III with VL, eight had a grade IIb, while one patient had grade I with VL. This patient had extremely small mouth opening and despite the good view with both DL (C/L IlIa) and VL (C/L I), manipulations of the tube within the oral cavity was very challenging. In this case, the thinner boogie was used as a guidewire to lead the tube to the trachea.

### 3.3. Relation between Prognostic Risk Factors and IDS

According to the univariate analysis (Spearman correlation), no significant relationships were found between cervical spine movement, interincisor gap, back teeth, ULBT score and BMI score, and the IDS.

### 3.4. Relation between VL View and IDS

Spearman correlation revealed that there was a strong positive correlation between IDS and the view with VL, which was statistically significant rs = 0.841, *p* < 0.0001.

## 4. Discussion

The present study confirmed the findings of previous studies, regarding the improvement of laryngoscopic view [6,18]. Aziz et al. demonstrated significantly better Cormarck–Lehane views with Storz^®^ C-MAC™, compared with direct laryngoscopy, in patients with at least one predictor of difficult intubation [18]. A recent meta-analysis showed that in the predefined subgroup of patients with difficult airway settings, glottic visualisation during tracheal intubation with the Storz^®^ C-MAC™ was superior to those with the Macintosh laryngoscope [6]. However, there is an inconsistency in literature regarding the definition of predicted difficult intubation. Aziz et al. used preoperative predictors of difficult intubation. Regardless of the presence of these criteria, most of their sample population had easy Laryngoscopic views (Cormack–Lehane view grades I or II) irrespective of whether the Storz^®^ C-MAC™ or standard DL was used [19]. In their meta-analysis, contrarily, Hoshijima et al. authors enrolled trials with different scenarios like cervical immobility, obesity, or lateral position [6]. By design, our study included only one cohort, undergone laryngoscopy with both DL and VL and as airway difficulty criteria used the classic difficult intubation prognostic factors. Our results both confirmed the higher proportion of patients with a Cormarck–Lehane III or IV view with DL as well as the improvement of laryngoscopic view with VL.

Despite the fact that all difficult airway algorithms recommend the use of fiberoptic intubation and preservation of spontaneous breathing in patients with known or suspected difficult airways, the definition of “suspected difficult airway” is still quite unclear. Apart from cases with known difficult or failed intubation, as well as cases with overt difficult laryngoscopy (cervical stabilization, lateral position, and airway pathology), the decision on whether awake fiberoptic or conventional laryngoscopy should be chosen, is often difficult to make. Furthermore, all preoperative difficult intubation predictors examined during conventional intubation, have been proven of low predictive value. A recent meta-analysis examined bedside predictors such as Mallampati score, thyromental distance, upper lip bite test, interincisor gap, and sternomental distance concluded that current evidence suggests these tests to have limited and inconsistent capacity to discriminate between patients with difficult and easy airways. Hence, the possibility of facing a non-anticipated difficult laryngoscopy and intubation scenario is still present.

Considering cases of failed intubation with DL, VL has been found to be the most frequently chosen rescue technique, with success rates of 92% for Storz^®^ C-MAC™ [20]. This high success rate observed, offers a significant margin of safety in cases where while a plan of conventional laryngoscopy with abolishment of spontaneous breathing had been chosen but intubation was proven challenging. Nevertheless, despite its high success rate, the use of Storz® C-MAC™ could not fully eliminate the possibility of failed intubation. Among the aims of this study was the evaluation of correlation between the classic preoperative bedside difficult intubation predictors and the improvement of laryngoscopic view. The selection of this outcome measure was made in order to investigate whether preoperative bedside difficult intubation predictors were more reliable during VL, compared to DL, making the decision on choosing between flexible awake or VL easier. Our analysis showed that among bedside risk factors examined, Mallampati III or IV and thyromental distance <6 cm were related to significant improvement of the laryngoscopic view. However, multivariate analysis showed that the discriminative value of these predictors was very poor (ROC-AUC = 0.57). Consequently, Storz® C-MAC™ significantly improves the view of patients with Mallampati III-IV or low thyromental distance, but the discriminative power or their combination is considered rather poor.

A weak point in our design is our choice not to follow a randomized, controlled crossover methodology and subject all patients to the same sequence of events, i.e., DL view followed by VL view and VL intubation. The reason for this decision was based on ethical concerns. Taking into consideration that our cohort included patients with at least two predictors of difficult intubation and that most studies tend to confirm the improvement of intubation with the use of VL, we decided to intubate the patients with the use of VL, minimizing intubation attempts to up to three. This design obviously precluded the possibility to draw conclusions about the easiness of intubation with DL in enrolled patients and the comparison with VL. One point of criticism regarding the use of video laryngoscopes is their “true” ability to improve intubation success rate apart from improvement of laryngoscopic view [21]. Despite these concerns, recent meta-analysis have demonstrated the superiority of VL compared to DL in difficult airway situations [6]. While direct conclusions about the superiority of VL could not be made from our study, VL was related with success rates up to 99%.

The high success rate of intubation with VL, in comparison with other studies [20] may be attributed to the patient population. Our eligibility criteria excluded patients with cervical spine or airway pathology. Albeit this fact, the present observational study revealed that even when multiple prognostic difficult intubation risk factors are present, the use of Storz® C-MAC™ possibly could lead to high success rates, further eliminating the discriminative power of these factors.

With regard to ease of intubation, IDS was independently correlated with the C-MAC™ VL view. Hence, apart from the high successful intubation rate, laryngoscopic view seemed to play a pivotal role in the difficulty in intubation. This is possibly more important in video laryngoscopies with conventional Macintosh blades and not in video laryngoscopes with extra-curved blades [19].

The advantage of the C-mac™ D-blade is based on the acute angle of vision. However, the C-mac™ D-blade has been related to more time-consuming manipulations. Even more, all acute angle laryngoscopes (such as C-mac™ D-Blade and Glidescope™) have raised questions about the failure to intubate, despite the good visualization of trachea. By design, intubation could not be compared for both laryngoscopes in our study. Hence, we preferred to use the classic C-mac™ and not C-mac™ D-blade in order to apply similar maneuvers and reduce the possibility of failed intubation despite good visualization.

## 5. Conclusions

In conclusion, C-MAC™ VL was proven to be faster and lead to better laryngoscopic views, compared to conventional DL, in patients with two or more preoperative risk factors for difficult intubation. Furthermore, among risk factors examined, only Mallampati score III or IV and thyromental distance ≤6 cm, were independently related to improvement of the laryngoscopic view. However, multivariate analysis showed poor prognostic value.

## Figures and Tables

**Table 1:** Patients’ characteristics.

Variables	Patients (N = 178)
Age (years), mean (S.D)	46.61 (18.61)
Men, *n* (%)	81 (45.5)
Women, *n* (%)	97 (54.5)
Weight (Kg), mean (S.D)	85.30 (17.11)
Height (m), mean (S.D)	1.63 (.09)
BMI (Kg/m^2^), mean (S.D)	29.50 (5.34)

**Table 2:** Correlation coefficients between risk factors with Cormack–Lehane scale’s improvement.

Variables	N	Odds Ratio (95% CI)	*p*-Value
Mallampati airway		2.5 (1.30, 5.08)	0.006 *
class 1–2	63		
class 3–4	113		
Thyromental distance		0.26 (0.05, 1.19)	0.05
≤6 cm	156		
>6 cm	20		
Cervical spine movement		0.81 (0.41, 1.59)	0.54
≤15 degrees	67		
>15 degrees	109		
Interincisor gap		0.95 (0.48, 1.88)	0.90
≤4 cm	105		
>4 cm	71		
Back teeth		1.13 (0.34, 3.71)	0.83
>0.5 cm	16		
≤0.5 cm	160		
ULBT score		1.61 (0.61, 4.08)	0.32
class 1–2	146		
class 3–4	30		
BMI		1.02 (0.41, 2.54)	0.96
<30 kg/m^2^	106		
>30 kg/m^2^	70		

* significantly related, Pearson’s chi-squared test.
